# Explaining the variation in the attained power of a stepped-wedge trial with unequal cluster sizes

**DOI:** 10.1186/s12874-020-01036-5

**Published:** 2020-06-24

**Authors:** Yongdong Ouyang, Mohammad Ehsanul Karim, Paul Gustafson, Thalia S. Field, Hubert Wong

**Affiliations:** 1 School of Population and Public Health, University of British Columbia, 2206 E Mall, Vancouver, BC V6T 1Z3 Canada; 2 Centre for Health Evaluation and Outcome Sciences, 588–1081 Burrard Street, St Paul’s Hospital, Vancouver, BC V6Z 1Y6 Canada; 3 Department of Statistics, University of British Columbia, 3182 Earth Science Building, 2207 Main Mall, Vancouver, BC V6T 1Z4 Canada; 4 Vancouver Stroke Program, Faculty of Medicine, University of British Columbia, S169–2211 Wesbrook Mall, Vancouver, BC V6T 2B5 Canada

**Keywords:** Cluster randomized trial, Power distribution, Treatment-time period correlation, Treatment group imbalance

## Abstract

**Background:**

In a cross-sectional stepped-wedge trial with unequal cluster sizes, attained power in the trial depends on the realized allocation of the clusters. This attained power may differ from the expected power calculated using standard formulae by averaging the attained powers over all allocations the randomization algorithm can generate. We investigated the effect of design factors and allocation characteristics on attained power and developed models to predict attained power based on allocation characteristics.

**Method:**

Based on data simulated and analyzed using linear mixed-effects models, we evaluated the distribution of attained powers under different scenarios with varying intraclass correlation coefficient (ICC) of the responses, coefficient of variation (CV) of the cluster sizes, number of cluster-size groups, distributions of group sizes, and number of clusters. We explored the relationship between attained power and two allocation characteristics: the individual-level correlation between treatment status and time period, and the absolute treatment group imbalance. When computational time was excessive due to a scenario having a large number of possible allocations, we developed regression models to predict attained power using the treatment-vs-time period correlation and absolute treatment group imbalance as predictors.

**Results:**

The risk of attained power falling more than 5% below the expected or nominal power decreased as the ICC or number of clusters increased and as the CV decreased. Attained power was strongly affected by the treatment-vs-time period correlation. The absolute treatment group imbalance had much less impact on attained power. The attained power for any allocation was predicted accurately using a logistic regression model with the treatment-vs-time period correlation and the absolute treatment group imbalance as predictors.

**Conclusion:**

In a stepped-wedge trial with unequal cluster sizes, the risk that randomization yields an allocation with inadequate attained power depends on the ICC, the CV of the cluster sizes, and number of clusters. To reduce the computational burden of simulating attained power for allocations, the attained power can be predicted via regression modeling. Trial designers can reduce the risk of low attained power by restricting the randomization algorithm to avoid allocations with large treatment-vs-time period correlations.

## Background

**Fig. 1:**
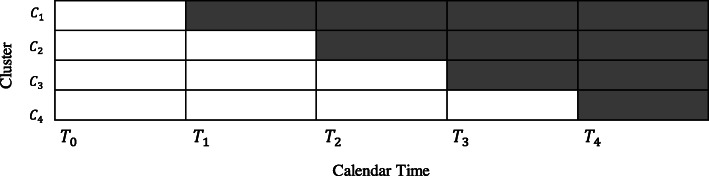
Diagram for a standard stepped-wedge cluster randomized controlled trial design. Cells in white correspond to periods during which participants receive standard-of-care, cells in grey correspond to periods during which participants receive the new intervention

Use of the stepped-wedge cluster randomized controlled trial (SW-CRT) design has increased dramatically in recent years [1–3]. In a standard stepped-wedge design (Fig. 1), every cluster (*C*_1_, *C*_2_, *C*_3_, *C*_4_) begins (time-point *T*_0_) with delivery of the existing standard-of-care (shown as white cells) to participants.

Clusters then transition to delivery of a new intervention (shown as gray cells) at randomly determined time-points (*T*_1_, …, *T*_4_), until all clusters are delivering the new intervention. The SW-CRT design often is used when there is a desire or need to implement and evaluate the intervention at the population level [4], or when it would not be logistically feasible to implement the intervention in every cluster at the same time [3], or when recruitment of clusters could be enhanced by ensuring all clusters eventually receive the new intervention. It has been implemented in trials exploring both single interventions as well as pathways of care in multiple settings [5–8]. Copas et al. [9] described three main SW-CRT designs: the “closed cohort”, the “open cohort”, and the “continuous recruitment short exposure” designs. In the first two designs, the treatment received by a participant during follow-up matches the treatment being delivered by his/her cluster at each time-point and each participant contributes multiple outcome measurements over time. In the third design, participants receive the treatment being delivered by his/her cluster at the time of study entry, remains on this treatment throughout follow-up, and contributes a single outcome measurement. For simplicity, we will refer to the continuous recruitment short exposure design as a “cross-sectional” design, a term which although less precise, is more commonly used [3, 10]. The results presented throughout this paper were derived using a cross-sectional SW-CRT.

Methods for calculating power and sample size in SW-CRTs with equal cluster sizes have been discussed widely in the literature [2, 11–14]. Code for calculating the power of a SW-CRT with equal cluster sizes are also available for commonly used statistical software (R [15, 16], Stata [17] and SAS [18]). Although it has been recognized that unequal cluster sizes in a parallel cluster randomized trial (P-CRT) leads to loss of power and efficiency [19, 20], limited work has been conducted on power and sample size calculations in unequal cluster size SW-CRTs. Hussey and Hughes [2] described a procedure for calculating approximate power based on a Wald test which allows for unequal cluster sizes in the SW-CRT, but they did not explore the impact of unequal cluster sizes. Kristunas et al. [21] conducted simulations that showed that the power of a SW-CRT with unequal cluster sizes was only slightly lower than if the cluster sizes had been equal. Girling [22] extended the method for evaluating the precision of regular single-period P-RCT with unequal cluster sizes to the stepped wedge design. Harrison et al. [23] derived first-order approximation formulae for the expected treatment effect variance given either known cluster sizes or the mean and coefficient of variation of the cluster sizes. They then used this treatment effect variance formula in a Wald test to conduct sample size calculations as well as to investigate the relative efficiency comparing equal and unequal cluster size SW-CRT designs and the design effect relative to individually randomized designs. However, it is important to recognize that these results focused on the *expected power*, that is, the power obtained through averaging over both 1) the randomness in the allocation of the clusters and 2) the random outcome variation across participants. Distinguishing between the two components is important. The latter source of variation is an intrinsic attribute of the population and outside the control of the investigator. However, the trial investigator has control over the former source through the choice of the randomization algorithm, and this choice impacts on trial power.

Both Wong et al. [24] and Martin et al. [25] showed that the *attained power* (Wong et al.), also called the *actual power* (Martin et al.), defined as the power associated with a specific allocation, can vary substantially across realized allocations and can be much lower than the expected power. Through deriving an analytic approximation, Matthews [26] showed how the variance of the treatment effect estimate varies across allocations. Thus, a trial that was expected to achieve a specified power prior to allocation of the clusters might turn out to be underpowered due to an “unlucky” allocation. For example, we are involved in a SW-CRT [27] with 20 hospitals (clusters) in which the two largest hospitals were expected to enrol more than 200 participants each, while the four smallest hospitals were expected to enrol less than 10 participants each, with a cluster size coefficient of variation of 1.17. Power calculations obtained via simulation showed that although using an unrestricted randomization algorithm would yield an expected power of 80%, the attained power varied from a low of 68% to a high of 83% depending on the allocation.

Martin et al. [25] observed larger variation in attained powers in designs with a smaller number of clusters, and a smaller intraclass correlation coefficient (ICC)*.* In addition, they observed that the absolute difference in the numbers of participants allocated to the two treatment groups, referred to hereafter as the “treatment group imbalance (TGI)”, explained only a small portion of the variability in attained power across allocations in the SW-CRT and that contrary to intuition, the attained power was relatively higher for allocations with a large TGI. In limited scenarios, Wong et al. [24] found that the (Pearson) correlation between the participant-level treatment assignment and the time-period with clustering ignored, referred to hereafter as the “treatment-vs-time period correlation (TTC)”, had a large impact on the attained power. They observed that the allocations with the greatest attained power were those in which the large clusters transitioned during the first or last steps (equally split) and that these allocations had the lowest TTC. In the SW-CRT, it is important to adjust for the time period to avoid bias in the treatment effect estimate because treatment group and time period inherently are highly correlated. They proposed that as TTC increases, this adjustment increases the standard error of the estimated treatment effect, which leads to a greater loss in attained power. However, we are not aware of a comprehensive study on these topics.

In this study, we investigated how different allocation characteristics interacted with design factors to affect attained power and the risk of obtaining low attained power in cross-sectional SW-CRTs. This risk can be assessed by constructing the *pre-randomization power distribution (PD)* [24], defined as the distribution of attained powers obtained from all possible allocations that a randomization algorithm can generate. A good randomization algorithm will ensure that the risk of obtaining a low attained-power allocation is acceptably small. Identifying such an algorithm requires an understanding of what factors cause low attained power. The first aim of this work was to gain an understanding of the factors that affect the risk of low attained power. We used simulation to evaluate the attained power across different allocations under a wide variety of scenarios. While it is possible to assess the attained power using approximate analytic formulae (e.g., Hussey & Hughes [2]), the accuracy of those formulae has not been well investigated in the context of SW-CRTs, especially when the number of clusters or the cluster sizes are low. However, the computational time needed to simulate the attained powers for all possible allocations often is not feasible. Hence, our second aim was to develop regression models that could accurately predict the attained power for any allocation based on allocation characteristics (specifically TGI and TTC) and the attained powers from a sample of allocations. The results of this work provide guidance on how to assess attained power and avoid having an unacceptably low attained power when designing a SW-CRT with unequal cluster sizes.

## Methods

### Aim one: evaluating attained power and the impact of different factors on the risk of low attained power

In this study, we assumed that individual outcomes, *Y*_*ijk*_, were generated from the following linear mixed-effects model:

1 $$ {Y}_{ij k}={\alpha}_i+\beta \times j+\theta \times {X}_{ij}+{\varepsilon}_{ij k} $$

where *i* indexed the cluster, *j* indexed the time period (*j* = 0 denotes baseline), and *k* indexed the individual within cluster *i* and time period *j*. The error terms *ε*_*ijk*_ were assumed to be independently sampled from a standard normal distribution (mean zero and variance ${\sigma}_e^2=1$). The treatment group indicator (0 = standard-of-care, 1 = intervention) was denoted as *X*_*ij*_ and the treatment effect, *θ*, was set to 0.26. As ${\sigma}_e^2=1$, the standardized effect size, *θ*/*σ*_*e*_, also equaled 0.26. Because the variance of the treatment effect estimate does not depend on the linear time effect coefficient, *β*, its value was set to 1 without loss of generality. (For simplicity, we assumed a fixed linear time effect rather than a separate fixed effect for each time period as assumed in the Hussey-Hughes model. This difference has no material impact on the findings of this work). The cluster-specific effects, *α*_*i*_, were assumed to be independently sampled from a normal distribution with mean zero and variance ${\sigma}_c^2$ . The value of ${\sigma}_c^2$ was calculated from set values of the ICC, where ICC was defined as $\frac{\sigma_c^2}{\sigma_c^2+{\sigma}_e^2}$. The treatment-vs-time period correlation (TTC) was defined as the Pearson correlation coefficient between treatment and the time-period, *corr*(*X*_*ij*_, *j*), across all individual observations with clustering ignored. The treatment group imbalance (TGI) was defined as the difference in the numbers of participants who received the new intervention versus the standard-of-care.

#### Computational challenge and a practical solution

Our aim was to obtain the set of attained powers by simulation and their corresponding power distribution for a specified randomization algorithm. First, we needed to obtain a list of all possible allocations that the randomization algorithm can generate, and then to evaluate the attained power associated with each possible allocation.

However, the evaluation of attained power for all potential allocations can be computationally challenging, given the potentially huge number of possible allocations. For example, a twenty-cluster cross-sectional SW-CRT with unique cluster sizes and four clusters transitioning at each of five steps has more than 300 billion unique allocations.

One strategy to reduce the number of unique allocations was by categorizing the clusters into size groups (e.g., (S)mall/(L) arge or (S)mall/(M)edium/(L)arge) and treating the clusters within each size group as identical, thereby reducing the number of unique allocations while increasing their multiplicities. Doing so will lead to only an approximate solution. However, this is often adequate in practice, given that the anticipated number of individuals that a cluster will enroll often can be only approximated at the start of a trial. In practice, we do not recommend having more than four size categories, as then the number of unique allocations likely will be too large to evaluate.

#### Simulation specifications

As shown in Table 1, we investigated the impact of the total number of clusters (12, 24 or 48), the number of cluster size groups (2 – S/L or 3 – S/M/L), the distribution of clusters across cluster size groups (equal or unequal), the coefficient of variation (CV) of the cluster sizes (0.4, 0.7, 1.0, and 1.3), defined as the ratio of standard deviation of cluster sizes to the mean, and the ICC of the response variable (0.01, 0.05, and 0.10). A full factorial layout was investigated for all of these factors except the CV. An equal distribution of clusters across cluster size groups corresponded to an equal number of clusters in each cluster size group; an unequal distribution corresponded to distribution of clusters in a S:L ratio of 3:1 for scenarios with two cluster size groups or 3:2:1 to S:M:L for scenarios with three cluster size groups. For unequal distributions, CVs of 0.4. 0,7, 1.0, and 1.3 were investigated for all unequal distribution scenarios, but for equal distribution scenarios, CVs of only 0.4 and 0.7 were investigated as a CV larger than one cannot be obtained with equal distributions. (Thus, when the actual cluster sizes have a CV larger than one, trial designers need to use unequal distributions to apply the approach presented here.) Thus, there were a total of 108 scenarios.

**Table 1:** Factors and their specific values explored in the simulation study

**Factor**	**Values**
**Number of clusters**	12, 24, 48
**Number of cluster size groups**	2, 3
**Distribution of clusters to cluster size groups**	Equal (6S + 6 L, 12S + 12 L, 24S + 24 L, 4S + 4 M + 4 L, 8S + 8 M + 8 L, 16S + 16 M + 16 L), Unequal (9S + 3 L, 18S + 6 L, 36S + 12 L, 6S + 4 M + 2 L, 12S + 8 M + 4 L, 24S + 16 M + 8 L)
**ICC**	0.01, 0.05, 0.1
**CV**	0.4, 0.7, 1.0^a^, 1.3^a^

^a^For equal distribution of clusters to cluster size groups, only CVs of 0.4 and 0.7 were investigated as a CV of 1 or larger is not possible

Trial parameter values for all 108 scenarios are listed in the Additional file (S.1). The number of transition steps was fixed at four. To determine the total number of individuals needed to obtain approximately 80% power in each scenario, we used Hussey and Hughes’s [2] sample size formula for a SW-CRT with equal cluster sizes as implemented by Baio et al. [11] in the R package “SWSamp [16]” (implementation of the Hussey and Hughes’s formula). Then, we re-distributed the total number of individuals to create unequal sized clusters that matched the CVs we set under each scenario (see Additional file, S.2 for details) [28]. If a resulting cluster size was not an integer, we set it at random to one of the two bracketing integers such that the expected value matched the initially calculated cluster size. For example, if the calculated cluster size was 12.3, the cluster size was set at random to either 12 or 13, such that its expected value was 12.3. Within a cluster, individuals were allocated to the time periods using a multinomial distribution with equal probability for each period. The total number of unique allocations is presented in the third column from the right in Additional file S.1. Among the 108 scenarios, 72 scenarios (referred to as ‘completed scenarios’) had less than 2000 unique allocations. For these scenarios, the attained power for every allocation was evaluated via simulation. For the other 36 scenarios (referred to as ‘sampled scenarios’), we evaluated the attained power for only a sample of 2000 allocations. Subsequently, the attained power for two sampled scenarios, one with a relatively small number of allocations (scenario #64, with 8623 allocations) and one with a relatively large number of allocations (scenario #103, with 113,949 allocations), had all of their allocations evaluated to enable validation of the prediction models that were constructed using only the original 2000 allocations.

#### Performance metrics

For each selected allocation, 10,000 datasets were simulated and analyzed using the model (1). Analysis was performed using the *lme* function in R [29]. This *P*-value is calculated through comparing the Wald statistic to quantiles from a *t*-distribution with degrees of freedom equivalent to what would be used in a balanced, multilevel ANOVA designs [30]. The attained power was estimated using the proportion of *P*-values less than 0.05. Assuming a power near 80%, the simulation standard error of each attained power was approximately 0.4%. The PD associated with the unrestricted randomization algorithm was then constructed from the estimated attained powers by weighting each attained power by the probability of the corresponding allocation. Two measures of the risk of low attained power were then obtained from the PD: (1) the probability that the attained power falls more than 5% below the expected power (obtained in the simulations), and (2) the probability that the attained power falls more than 5% below the nominal 80% power (i.e., less than 75%) that would be achieved in a trial with equal cluster sizes. The first measure provides an indication of whether potential low attained power is a concern when power is calculated using the approach presented here for handling unequal cluster sizes. The second measure would be of greater interest when one is assessing whether power calculations obtained under the assumption of equal cluster sizes are adequate. We set a 5% power loss as being meaningful, but trial designers may wish to choose a different value more relevant to their context. In this paper, the phrase “risk of low attained power” will be used as a short form to refer to either of these measures. All computations were conducted using R version 3.5.0 on Cedar, Compute Canada [31, 32].

### Aim two: explaining the variation in attained power across allocations and predicting attained power using allocation characteristics

Using the simulation results from Aim One, we first constructed scatterplots to examine the bivariate relationship between the simulated attained power and each of TTC and TGI separately. Then, to predict the attained power for each scenario, we fitted logistic regression models of the proportion of simulations in each allocation that achieved statistical significance (at level 0.05) as a function of linear and quadratic terms for TTC and TGI.

Based on the relationships observed in the bivariate scatterplots, we chose to investigate a sequence of four nested models. The terms included in the four models that were considered were: (Model 1: TTC), (Model 2: TTC, TGI), (Model 3: TTC, TGI, TTC^2^), and (Model 4: TTC, TGI, TTC^2^, TGI^2^).

We measured the predictive accuracy of each model by using five-fold cross-validation, conducted using cv.glm [33] in R, to estimate the root mean squared prediction error (RMSPE), defined by $RSMPE={\sum}_{k=1}^5\frac{n_k}{n}\left(\frac{\sum_{i\in {C}_k}{\left({p}_i-\hat{p_i}\right)}^2}{n_k}\right)$, where *C*_*k*_ represented the set of allocations in the *k*-th partition, *n*_*k*_ was the number of allocations in the *k*-th partition, *p*_*i*_ and $\hat{p_i}$ were the simulated and predicted powers, respectively, for the *i*-th allocation, and *n* was the number of unique allocations in the scenario. From these models, we selected the one for which no meaningful improvement in RMSPE was achieved by adding another term to the model. For the selected model, we examined additional predictive performance measures for each scenario, including maximum and average absolute prediction errors ($\mid {p}_i-\hat{p_i}\mid$). For two sampled scenarios (#64, #103), we validated the selected model with respect to both prediction of attained power for individual allocations, and estimation of the risk of low attained power. For the former objective, we compared the predicted attained power to the simulated attained power for each allocation that was not in the set used to fit the predictive model. For the latter objective, we repeatedly sampled (10,000 times) 2000 allocations and estimated the risk of low attained power from the fitted model. We then compared these estimated risks to the “true” risk derived from the simulated attained powers.

## Results

### Aim one: evaluating the attained powers and factors affecting the risk of low attained power

In the body of this paper, we report on the scenarios with an unequal distribution of clusters to cluster size groups. The results for scenarios with an equal distribution of clusters to cluster size groups were similar when matched on the remaining factors (see Additional file, S.3.1 and S.3.2).

**Fig. 2:**
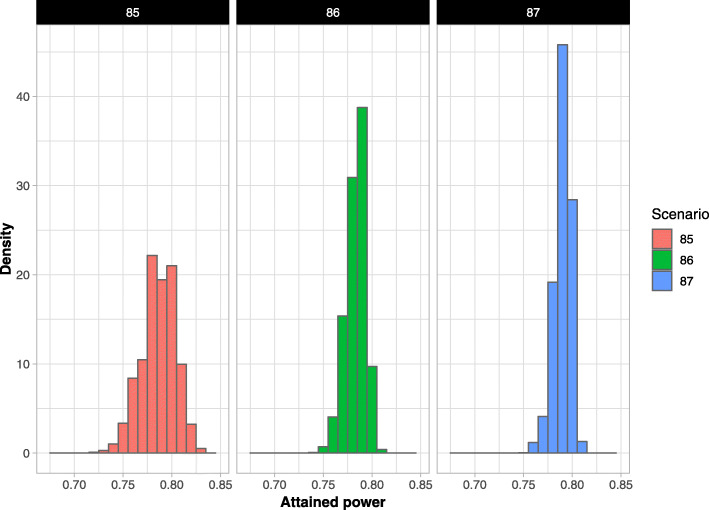
Examples of power distributions: scenario 85 (48 clusters: 36 L (size: 56.99), 12 S (size: 9); CV:1.0; ICC:0.01), scenario 86 (48 clusters: 36 L (size: 67.85), 12 S (size: 10.72); CV:1.0; ICC:0.05). scenario 87 (48 clusters: 36 L (size: 75.99), 12 S (size: 12); CV:1.0; ICC:0.1)

Substantial variation in attained powers was observed in all the scenarios. Across all simulations, attained power ranged from 0.62 to 0.86. As examples, the PDs for selected scenarios (#85 to #87) are displayed in Fig. 2. The expected powers obtained from the simulations (Additional file, S.1, the “Weighted expected power”) usually were quite close to the nominal 80% power associated with an equal cluster size design, but losses of up to roughly 4% were seen when the CV was large.

#### ICC

**Fig. 3:**
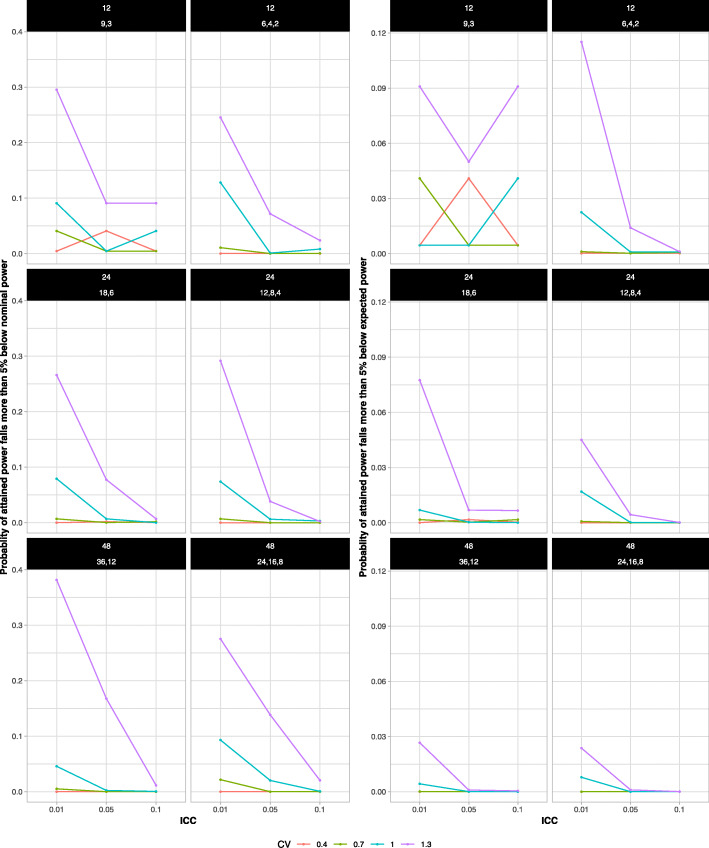
The probability that the attained power falls more than 5 % below the nominal (left set of panels) or the expected (right set of panels) power by ICC. The risks decreased as ICC increased in all scenarios, except for the ones with 12 clusters. Panel labels identify the total number of clusters (first line) and the distribution of clusters to the cluster size groups S:L or S:M:L (second line)

Figure 3 shows the risk of low attained power (for both measures) as a function of ICC for different combinations of the total number of clusters, the CV, and the distribution of clusters to the size groups. The risk of low attained power varied greatly with ICC. For both risk measures, with a total of 24 or 48 clusters, the risk of obtaining low power was smaller in the scenarios with larger ICCs. If nominal power is used as the reference, the risk was as high as 38% when the ICC was 0.01 but dropped to nearly zero even when the ICC was 0.1. When there were 12 clusters in total, the risk of low attained power often continued to be high even with larger ICCs, and the patterns were not as evident. However, it should be noted that in these scenarios, the number of unique allocations is quite low so the combined effect of simulation error and discretization effects may have disrupted the monotonic patterns that were expected.

#### Coefficient of variation

**Fig. 4:**
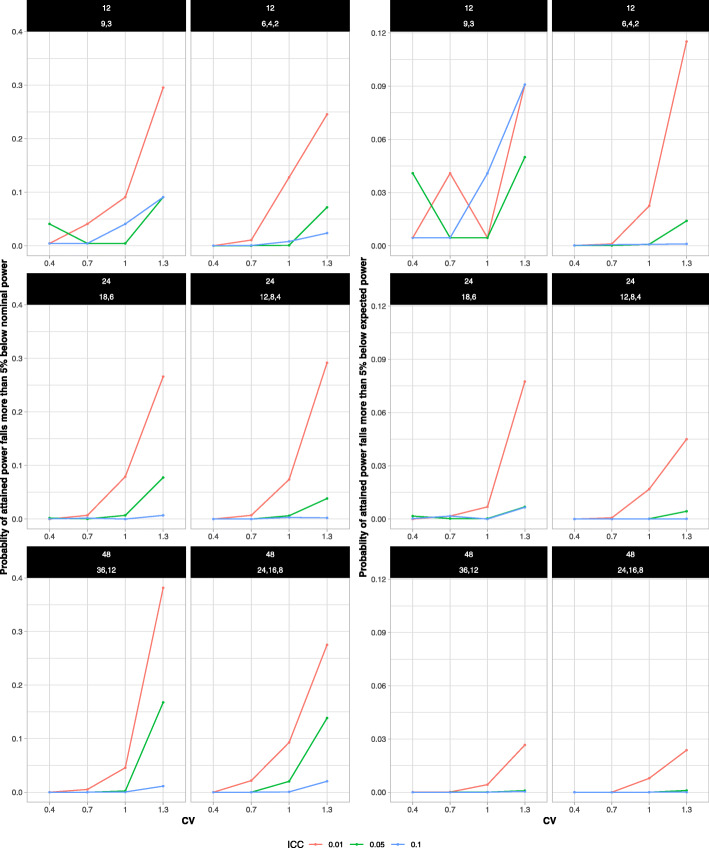
The probability that the attained power falls more than 5 % below the nominal (left set of panels) or the expected (right set of panels) power by CV. The risks increased as CV increased in all scenarios except the ones with 12 clusters. The risks were near zero when the CV was smaller than 0.7, except for the scenarios with 12 clusters and two size groups. Panel labels identify the total number of clusters (first line) and the distribution of clusters to the cluster size groups S:L or S:M:L (second line)

Figure 4 plots the risks of low attained power as a function of the CV. The risk of low attained power varied greatly across the CVs and depended on the ICC. Except for the scenarios with 12 clusters and two size groups, the risk was near zero when the CV was 0.4. However, as the CV increased above 0.7, the risk increased rapidly up to more than 30% when the CV was 1.3 and the ICC was 0.01.

### Aim two: explaining the variation in attained power across allocations and predicting attained power using allocation characteristics

#### Relationship among transition time-points of large clusters, treatment-vs-time period correlation (TTC) and absolute treatment group imbalance (TGI)

**Fig. 5:**
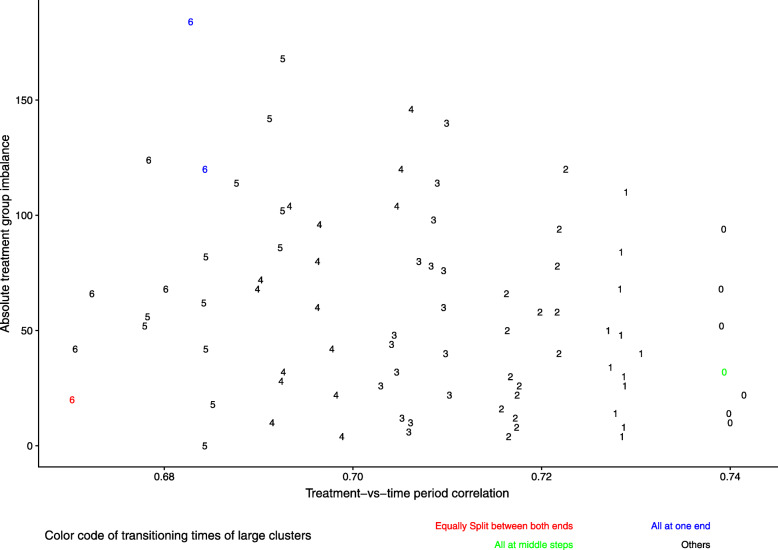
The relationship between the transition time-points of the large clusters with TTC and TGI from one simulation run for a selected scenario (scenario 37: 24 clusters with 18S,6L). The trend is typical for other runs. The plotted number indicates the number of large clusters transitioning at end steps for that allocation. When all of the large clusters transition at the middle steps (number in green), TTC is high and TGI is low. When all of the large clusters transition at one end step (number in red), both TTC and TGI are low. When all of the large clusters transition equally split between the two ends (number in blue), TTC is low while TGI is high

Understanding the relationship between the time-points when the large clusters transition, TTC, and TGI will be helpful for interpreting the predictive models presented later. Figure 5 summarizes the relationship typically seen between the transition time-points of the large clusters with TTC and TGI across simulation runs from one representative scenario (#37).

Plotted symbols represent different allocations. The number used as the plotting symbol indicates the number of large clusters transitioning at the two ends (i.e., at either the first or last step). Setting aside the values of TGI, this plot shows that the number of large clusters transitioning at the ends strongly determines TTC, with TTC decreasing as this number increases. When the transition time-points of the large clusters are equally split between the two ends, both TTC and TGI are low (numbers plotted in red), but when these transitioning times are concentrated at one end, TTC remains low while TGI is high (numbers plotted in blue). When all of the large clusters transition at the middle steps (numbers plotted in green), TGI will be low and TTC is high.

#### Impact of treatment-time period correlation on attained power

**Fig. 6:**
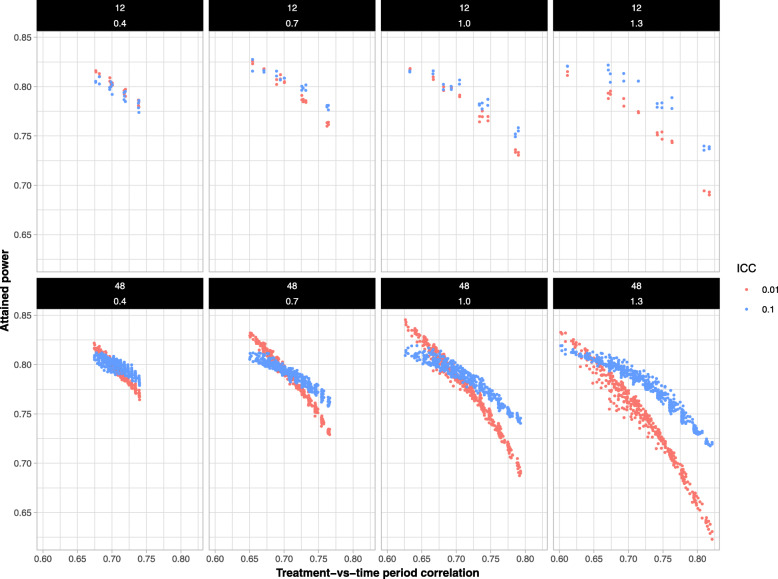
The relationship between treatment-vs-time period correlation and attained power among 16 scenarios. Columns represented difference CV values (from left to right: 0.4, 0.7, 1.0, 1.3). Rows correspond to the number of clusters (12 in the first row; 48 in second row). The colors of the plotted points correspond to ICC of 0.1 (blue) and ICC of 0.01 (red). The attained power decreases considerably as the treatment-vs-time period correlation increases

Figure 6 displays the relationship between the attained power for a given allocation and TTC for a selected set of 16 scenarios (two size groups, 12 or 48 clusters, ICC = 0.01 or 0.1). Within this grid of panels, columns correspond to different CV values (0.4, 0.7, 1.0, 1.3 from left to right) and rows correspond to the number of clusters (12 in the first row; 48 in the second row).

Within each panel, points in blue correspond to an ICC of 0.1, while those in red correspond to an ICC of 0.01. A graph for all scenarios is included in the Additional file, S.4.1 Across all scenarios, TTC consistently exhibited a strong negative, and mildly non-linear, relationship with the attained power. Greater variation in attained power was observed in the scenarios with a larger CV, a lower ICC, and a smaller number of clusters.

#### Impact of treatment group imbalance on attained power

**Fig. 7:**
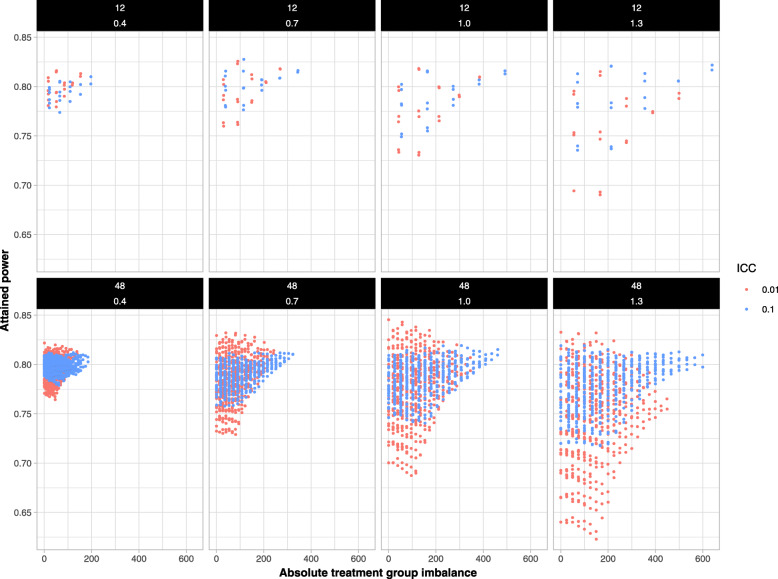
The relationship between treatment group imbalance and attained powers among 16 scenarios. Columns represented difference CV values (from left to right: 0.4, 0.7, 1.0, 1.3). Rows correspond to the number of clusters (12 in the first row; 48 in second row). The colors of the plotted points correspond to ICC of 0.1 (blue) and ICC of 0.01 (red). A triangle pattern was observed in most scenarios. Large treatment group imbalances appeared to be associated with higher power

Figure 7 displays the relationship between attained power and TGI, using the same layout and for the same scenarios used in Fig. 6. A graph for all scenarios is included in the Additional file, S.4.2 Across all scenarios, the association exhibited a “triangle” pattern with much weaker associations than were seen for TTC. In 90% (97/108) of the scenarios, univariate regression model fits showed that allocations with a large TGI were associated with a relatively higher attained power. This is counter-intuitive and opposite to the P-CRT setting, where attained power decreases with increasing TGI. When TGI was low, the attained power ranged over both low and high values across different allocations. This unexpected result is resolved in the following section. Essentially, the complicated relationship between TTC and TGI as described earlier leads to the true impact of TGI on attained power being confounded by its association with TTC, with the latter being a much stronger determinant of attained power.

#### Predictive model

Without any predictors (i.e., the null model with only an intercept term), the RMSPE of the predicted attained power ranged across the 108 scenarios from 0.0060 to 0.0454 with a median of 0.0157. When TTC alone (Model 1) was added to the model, the RMSPE decreased in every scenario with the median RMSPE shrinking down to 0.0037 (range: 0.0022, 0.0103). When TGI was then added (Model 2), the RMSPE decreased in 93 of the scenarios and the median RMSPE dropping down to 0.0035 (range 0.0020 to 0.0085). Note that in comparison to the crude effect of TGI, after adjustment for TTC, the percentage of scenarios with a positive TGI coefficient declined from 90 to 68% (74/108) and the distribution of TGI coefficients was compressed much closer to zero compared to the distribution of TGI coefficients from the unadjusted models (see Additional file, S.5).

On adding the TTC^2^ term (Model 3), the RMSPE decreased in 99 scenarios, with the median RMSPE shrinking to 0.0031 (range 0.0017, 0.0065). Further adding TGI^2^ (Model 4), resulted in the RMSPE decreased in 71 scenarios but the median RMSPE changed negligibly (i.e., on the order of 10^−5^). Hence, we selected the Model 3 as the final model.

**Fig. 8:**
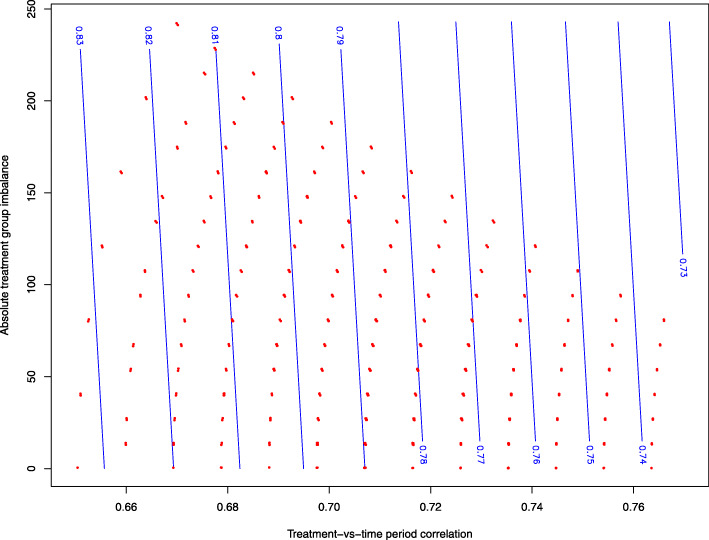
Contour plot of predicted attained power as a function of the treatment group imbalance and the treatment-time period correlation for scenario 79. The red dots correspond to the possible allocations in this scenario. The nearly vertical contour curves show that attained power is determined mainly by the treatment-time period correlation and the treatment group imbalance has only a small impact

Figure 8 displays a contour plot of the predicted attained power derived from the regression model for one scenario (#79).

The almost-vertical curves indicate that attained power decreased substantially as TTC increased. However, when TTC was fixed, the attained power decreased only slightly as TGI increased.

RMSPE, maximum absolute prediction error, and average absolute prediction error for each scenario are shown in S.6 in the Additional file.

#### Validation of prediction model

The medians of the differences between the predicted and the simulated attained powers for the allocations not used in model fitting for scenarios #64 and #103 were near zero and 95% of the predicted attained powers fell within 0.0069 (#64) and 0.0094 (#103) of the simulated attained powers (See Additional file, S.7 for the distribution of differences). This magnitude of prediction error is comparable to the expected error in the simulated attained powers (i.e., 95% of simulated attained power within 2 × 0.004 = 0.008), suggesting the selected model performs nearly as well as simulation. The true risks of low attained power (> 5% below nominal 80%), calculated from the simulated attained powers, were 0.0201 (#64) and 0.0697 (#103). In repeated sampling and fitting of the selected model, the median absolute difference between the model-based estimate and the true risk of low attained power was 0.0010 (#64) and 0.0019 (#103), with 95% of the values falling within 0.0018 (#64) and 0.0034 (#103).

## Discussion

### Understanding the risk of obtaining low power

Using simulation, we have examined how and why the attained power in an SW-CRT with unequal cluster sizes may be different from the expected power that typically is the focus of power calculations. We have argued that even if the expected power is adequate, the risk that the randomization leads to a trial with low attained power should be considered. The extension of the CONSORT 2010 statement [34] recommends that researchers report the unequal cluster sizes in trials. This study highlights the importance of knowing the cluster sizes to enable appropriate assessments of power. The power distribution constructed from the set of attained powers can be used to evaluate the risk that a given randomization algorithm will yield a trial with unacceptably low power.

As shown in our study, the CV of the cluster sizes strongly impacted the risk of low attained power. For CV values below 0.7, the risk of the attained power falling more than 5% below the expected power across the investigated ranges of values for the other parameters was sufficiently low that this risk may not be a concern. As the CV increased above 0.7, the risk of low attained power increased substantially, especially when the ICC was low. As the ICC increased, the risk of low attained power decreased. This result is consistent with Matthews et al. [35], who showed that in a “row-column” analysis of a SW-CRT, which corresponds to a linear mixed effects analysis, the variance of the treatment effect decreased as the ICC increased. Our heuristic explanation is that when the ICC was large, the *effective* sample sizes of large clusters were reduced greatly, which in turn reduced the *effective* CV of the cluster sizes. As expected, the risk of low attained power decreased as the number of clusters increased. However, even with 48 clusters, this risk was not ignorable when the CV was large, and the ICC was small.

### Explaining the variation in attained power

A key contribution of this study is clarifying how attained power is impacted by the joint effects of treatment-vs-time period correlation and absolute treatment group imbalance. TTC was shown to be the dominating factor. Our results suggest that the observation that TGI on its own is only weakly related to attained power and in the direction opposite to what is expected, made by Martin et al. [25] and confirmed here, is a consequence of confounding with TTC. After adjusting for TTC, the impact of TGI on the attained power was reduced or the direction reversed in nearly every scenario. That the direction was not reversed for every scenario may be due in part to simulation noise, but it could also reflect residual confounding due to the model mis-specifying the true dependence of attained power on TTC, or the omission of other important predictors.

We have shown that a model containing TTC, TTC^2^ , and TGI can accurately predicts attained power for any allocation, and hence to construct the power distribution for any randomization algorithm and to estimate the risk of low attained power, when the number of allocations is too large to evaluate their attained powers using simulation.

The original rationale for creating size groups was to reduce the number of unique allocations that needed to be evaluated to obtain the power distribution. However, given the high accuracy seen in these predictive models, creating cluster size groups may not be necessary. Instead, a predictive model could be built based on a representative sample of allocations without grouping of clusters and this model could be used to predict the attained powers associated with each non-sampled allocation. The caveats here are that some effort may be needed to determine how large a sample is needed to achieve a target predictive accuracy, and when the number of unique allocations is large, simply listing out all possible unique allocations may require excessive (computational) effort in which case size groups will still be needed.

Examination of attained power can assist in the identification of the allocation characteristics that lead to low attained power. For example, our study verified the results from previous work [24] showing that transitioning large clusters during the middle steps can increase the treatment-vs-time period correlation and lead to low attained power. This result is also consistent with Kasza and Forbes [36], who showed that for an equal-cluster-size design, the sequences (i.e., allocations) that transition during the middle steps contribute less information than those that transition at the end steps. Therefore, allocating large clusters to transition during the middle steps should (as a heuristic) yield less information than if they were allocated to transition at the end steps. Identifying these allocations affords trialists the opportunity to take pre-emptive actions to mitigate this problem. The simplest option would be to restrict the randomization algorithm to exclude low power allocations. However, this approach risks violating the criteria needed for valid randomization inference, as it could result in some clusters having no chance of being randomized to transition at particular time-points (e.g., large clusters may not be allowed to transition during the middle step(s)). A more conservative option could be to stratify randomization based on cluster size. This would ensure that every large cluster has a chance to be allocated to any transition step, while ensuring even distribution of large clusters across all of the steps. This would tend to yield a low-variability power distribution, as the excluded allocations would tend to be both those with low and high power. Further exploration of potential restricted randomization algorithms and their validity, benefits and limitations is needed.

### Limitations

The Wald test we used to evaluate statistical significance is known to yield higher-than-nominal Type I error rates [37]. Therefore, the powers that are reported here will tend to be higher than what would be obtained using a test that achieved the nominal size. We have no reason to expect that the patterns of findings that we have reported would be different if the test used achieved the nominal size. However, important future work would be to ascertain which test(s) provide the most accurate size and power values to ensure that real-life trial designs fulfill the desired statistical criteria. Some work towards this goal has been conducted by Tanner [38], using various analytic models (GLMM, GEE, cluster-level analysis) for binary outcomes.

Our study left several design factors as fixed values, including the number of transition steps at four, and a standardized effect size of 0.26. Others [25] have reported that the trial power is only weakly affected by the number of steps. Results from limited simulations we conducted with six steps were concordant with those reports (data not shown), so we did not pursue a comprehensive investigation in this regard. The standardized effect size of 0.26 arose from a calculation performed to achieve 80% power in one particular scenario. We adopted this value as being within the typical range seen in real trials [39]. Assuming a larger effect size would have led to smaller cluster sizes needed to achieve 80% power. Again, we have no reason to expect different patterns to our findings, but smaller sample sizes may lead to greater Type I error and inaccuracy in the estimated power. The numbers of clusters (12/24/48) were chosen primarily to reflect our belief that these are typical numbers seen in real SW-CRTs (but also for convenience as these choices allowed the numbers of clusters to be balanced across steps and cluster size groups). While we found that increasing the number of clusters reduces the risk of low attained power, we did not establish a bound above which this risk is ignorable. When the CV is large and the ICC is small, this risk will need to be evaluated even when a trial includes a larger number of clusters.

Due to limits on available computational time, we validated the predictive model for only two scenarios. The selected predictive model was shown to predict the attained power with accuracy similar to that achieved using simulation with 10,000 replicates. However, because this model may be mis-specified, there is no guarantee that this model will yield sufficiently accurate predictions if greater precision is required. Alternative models may need to be developed, perhaps including other allocation characteristics. In addition, simulating attained power for 2000 allocations required substantial computational time. An attractive goal would be developing accurate analytic formulae, like the one derived by Matthews [26] for the treatment effect variance, to support faster calculation of attained power.

This work considered only a continuous outcome within a cross-sectional SW-CRT. Further work is needed to extend the results to other types of outcomes (binary, count, survival, etc.) and to other SW-CRT designs.

## Conclusion

In a stepped-wedge trial with unequal cluster sizes, the risk that randomization yields an allocation with inadequate attained power is a function of the ICC, the CV of the cluster sizes, and the total number of clusters. To reduce the computational burden of simulating attained power for every allocation, the attained power can be predicted from the treatment-vs-time period correlation and the treatment group imbalance via regression modeling. Trial designers can reduce the risk of low attained power by restricting the randomization algorithm to reduce the chance of obtaining an allocation which yields a large treatment-vs-time period correlation.

## Supplementary information

**Additional file 1: S.1** List of all evaluated scenarios. **S.2** Cluster size re-distribution Calculation. **S.3.1** Risk of obtaining low vs ICC for scenarios with equal distribution of clusters. **S.3.2** Risk of obtaining low vs CV for scenarios with equal distribution of clusters. **S.4.1** The relationship between TTC and attained power for all scenarios. **S.4.2** The relationship between TGI and attained power for all scenarios. **S.5** Distribution of the coefficient for TGI before and after adjusting for TTC. **S.6** The RMSPE, maximum absolute prediction error, and average absolute prediction error for all scenarios. **S.7** Side-by-side violin plot showing the distribution of difference between the predicted and simulated attained powers for the allocations not used in model fitting for scenario #64 and #103.

## Data Availability

Not applicable. Simulation codes are available from: https://github.com/douyangyd/swcrtpd

## Abbreviations

CRT: Cluster randomized trial
CV: Coefficient of variation
ICC: Intraclass correlation coefficient
P-CRT: Parallel-arm cluster randomized trial
PD: (Pre-randomization) power distribution
RMSPE: Root mean square prediction error
SW-CRT: Stepped-wedge cluster randomized trial
TGI: Treatment group imbalance
TTC: Treatment-vs-time period correlation

## References

[1] Hemming K, Haines TP, Chilton PJ (2015). The stepped wedge cluster randomised trial: rationale, design, analysis, and reporting. BMJ.

[2] Hussey MA, Hughes JP (2007). Design and analysis of stepped wedge cluster randomized trials. Contemp Clin Trials.

[3] Brown CA, Lilford RJ (2006). The stepped wedge trial design: a systematic review. BMC Med Res Methodol.

[4] Grayling MJ, Wason JMS, Mander AP (2017). Stepped wedge cluster randomized controlled trial designs: a review of reporting quality and design features. Trials.

[5] Durovni B, Saraceni V, Moulton LH (2013). Effect of improved tuberculosis screening and isoniazid preventive therapy on incidence of tuberculosis and death in patients with HIV in clinics in Rio de Janeiro, Brazil: a stepped wedge, cluster-randomised trial. Lancet Infect Dis.

[6] Bacchieri G, Barros AJD, dos Santos JV (2010). A community intervention to prevent traffic accidents among bicycle commuters. Rev Saude Publica.

[7] Tirlea L, Truby H, Haines TP. Investigation of the effectiveness of the “girls on the go!” program for building self-esteem in young women: trial protocol. Springerplus. 2Epub ahead of print 19 December 2013. 10.1186/2193-1801-2-683. 10.1186/2193-1801-2-683 PMC3877412 24386627

[8] Gruber JS, Reygadas F, Arnold BF (2013). A stepped wedge, cluster-randomized trial of a household UV-disinfection and safe storage drinking water intervention in rural Baja California Sur, Mexico. Am J Trop Med Hyg.

[9] Copas AJ, Lewis JJ, Thompson JA (2015). Designing a stepped wedge trial: three main designs, carry-over effects and randomisation approaches. Trials.

[10] Barker D, McElduff P, D’Este C (2016). Stepped wedge cluster randomised trials: a review of the statistical methodology used and available. BMC Med Res Methodol.

[11] Baio G, Copas A, Ambler G (2015). Sample size calculation for a stepped wedge trial. Trials.

[12] Hemming K, Taljaard M (2016). Sample size calculations for stepped wedge and cluster randomised trials: a unified approach. J Clin Epidemiol.

[13] Woertman W, de Hoop E, Moerbeek M (2013). Stepped wedge designs could reduce the required sample size in cluster randomized trials. J Clin Epidemiol.

[14] Zhou X, Liao X, Spiegelman D (2017). “Cross-sectional” stepped wedge designs always reduce the required sample size when there is no time effect. J Clin Epidemiol.

[15] Hughes J, Hakhu NR, Voldal E. swCRTdesign: stepped wedge cluster randomized trial (SW CRT) design, https://cran.r-project.org/web/packages/swCRTdesign/index.html. (Accessed 20 Aug 2019).

[16] Baio G, Leech R. SWSamp: Computes Sample Size for a Stepped Wedge Design, using Simulation-Based Calculations., R package version 0.3. 2018. http://www.statistica.it/gianluca/software/swsamp/ (Accessed 13 May 2019).

[17] Hemming K, Girling A (2014). A menu-driven Facility for Power and Detectable-Difference Calculations in stepped-wedge cluster-randomized trials. Stata J.

[18] Teerenstra S, Taljaard M, Haenen A (2019). Sample size calculation for stepped-wedge cluster-randomized trials with more than two levels of clustering. Clin Trials.

[19] Eldridge SM, Ashby D, Kerry S (2006). Sample size for cluster randomized trials: effect of coefficient of variation of cluster size and analysis method. Int J Epidemiol.

[20] van Breukelen GJP, Candel MJJM (2012). Efficiency loss because of varying cluster size in cluster randomized trials is smaller than literature suggests. Stat Med.

[21] Kristunas CA, Smith KL, Gray LJ (2017). An imbalance in cluster sizes does not lead to notable loss of power in cross-sectional, stepped-wedge cluster randomised trials with a continuous outcome. Trials.

[22] Girling AJ (2018). Relative efficiency of unequal cluster sizes in stepped wedge and other trial designs under longitudinal or cross-sectional sampling. Stat Med.

[23] Harrison LJ, Chen T, Wang R. Power calculation for cross-sectional stepped wedge cluster randomized trials with variable cluster sizes. Biometrics. 10.1111/biom.13164. 10.1111/biom.13164 PMC7165039 31625596

[24] Wong H, Ouyang Y, Karim ME (2019). The randomization-induced risk of a trial failing to attain its target power: assessment and mitigation. Trials.

[25] Martin JT, Hemming K, Girling A (2019). The impact of varying cluster size in cross-sectional stepped-wedge cluster randomised trials. BMC Med Res Methodol.

[26] Matthews JNS. Highly efficient stepped wedge designs for clusters of unequal size. Biometrics. 2020. 10.1111/biom.13218. 10.1111/biom.13218 31961447

[27] ClinicalTrials.gov [Internet] Ho K, University of British Columbia,. Identifier NCT03439384, TEC4Home heart failure: using home health monitoring to support the transition of care; 2018, 2020. Mar 24 [cited 2020 Apr 13]; [about 6 screens]. Available from https://clinicaltrials.gov/ct2/show/NCT03439384.

[28] Hasselman B. Nleqslv: Solve Systems of Nonlinear Equations., R package version 3.3.2; 2018. https://cran.r-project.org/package=nleqslv (Accessed 12 Nov 2019).

[29] Pinheiro J, Bates D, DebRoy S, Sarkar D, Core Team R (2019). nlme: Linear and nonlinear mixed effects models. R package version.

[30] Pinheiro JC, Bates DM. Theory and computational methods for linear mixed-effects models. In: Mixed-effects models in S and S-PLUS. New York: Springer; 2000. p. 57–96.

[31] R Development Core Team. R: a language and environment for statistical computing. Vienna: R Foundation for Statistical Computing; 2008. ISBN 3–900,051–07-0, URL http://www.R-project.org. Accessed 15 Mar 2019.

[32] Compute Canada Cedar - CC Doc, https://docs.computecanada.ca/wiki/Cedar. (Accessed 1 May 2019).

[33] Canty A, Ripley B. Boot: bootstrap functions (originally by Angelo Canty for S)https://CRAN.R-project.org/package=boot. (Accessed 28 Mar 2020); 2019.

[34] Hemming K, Taljaard M, McKenzie JE (2018). Reporting of stepped wedge cluster randomised trials: extension of the CONSORT 2010 statement with explanation and elaboration. BMJ.

[35] Matthews JNS, Forbes AB (2017). Stepped wedge designs: insights from a design of experiments perspective. Stat Med.

[36] Kasza J, Forbes AB (2019). Information content of cluster–period cells in stepped wedge trials. Biometrics.

[37] Johnson JL, Kreidler SM, Catellier DJ (2015). Recommendations for choosing an analysis method that controls type I error for unbalanced cluster sample designs with Gaussian outcomes. Stat Med.

[38] Tanner W. Improved Standard Error Estimation for Maintaining the Validities of Inference in Small-Sample Cluster Randomized Trials and Longitudinal Studies. Theses and Dissertations--Epidemiology and Biostatistics. Epub ahead of print 1 January 2018. 10.13023/etd.2018.434.

[39] Rothwell JC, Julious SA, Cooper CL (2018). A study of target effect sizes in randomised controlled trials published in the health technology assessment journal. Trials.

