# Supplementary material for: Explaining the variation in the attained power of a stepped-wedge trial with unequal cluster sizes
Source: BMC Med Res Methodol. 2020 Jun 24;20:166. doi: 10.1186/s12874-020-01036-5 (PMC7315519; doi:10.1186/s12874-020-01036-5)

Additional file

S.1 List of all evaluated scenarios

Scen.	Nclus	Nclus type	Cluster size (S,L or S, M, L)	CV	ICC	Power from SWSamp	Number of unique allocations	Weighted expected power	Loss of power
**1**	12	9,3	75.16, 160.51	0.4	0.01	0.8002	20	0.7996	0.0006
**2**	12	9,3	91.91, 196.27	0.4	0.05	0.7997	20	0.8029	-0.0033
**3**	12	9,3	96.19, 205.42	0.4	0.1	0.8003	20	0.7947	0.0055
**4**	12	6,6	59.54, 133.46	0.4	0.01	0.8002	44	0.7998	0.0004
**5**	12	6,6	72.81, 163.19	0.4	0.05	0.7997	44	0.7999	-0.0002
**6**	12	6,6	76.2, 170.8	0.4	0.1	0.8003	44	0.7994	0.0008
**7**	12	9,3	59.16, 208.52	0.7	0.01	0.8002	20	0.7954	0.0048
**8**	12	9,3	72.34, 254.98	0.7	0.05	0.7997	20	0.7994	0.0004
**9**	12	9,3	75.71, 266.86	0.7	0.1	0.8002	20	0.8040	-0.0038
**10**	12	6,6	31.83, 161.17	0.7	0.01	0.8002	44	0.7874	0.0128
**11**	12	6,6	38.92, 197.08	0.7	0.05	0.7997	44	0.7956	0.0041
**12**	12	6,6	40.73, 206.27	0.7	0.1	0.8003	44	0.7955	0.0048
**13**	12	9,3	43.16, 256.53	1	0.01	0.8002	20	0.7821	0.0181
**14**	12	9,3	52.77, 313.68	1	0.05	0.7997	20	0.7946	0.0051
**15**	12	9,3	55.23, 328.3	1	0.1	0.8003	20	0.7895	0.0108
**16**	12	9,3	27.15, 304.54	1.3	0.01	0.8002	20	0.7657	0.0345
**17**	12	9,3	33.2, 372.39	1.3	0.05	0.7997	20	0.7897	0.0100
**18**	12	9,3	34.75, 389.74	1.3	0.1	0.8003	20	0.792	0.0083
**19**	12	6,4,2	66.16, 105.75, 169.04	0.4	0.01	0.8002	238	0.7947	0.0055
**20**	12	6,4,2	80.9, 129.31, 206.7	0.4	0.05	0.7997	238	0.8009	-0.0012
**21**	12	6,4,2	84.67, 135.34, 216.33	0.4	0.1	0.8003	238	0.7999	0.0003
**22**	12	4,4,4	55.19, 89.42, 144.88	0.4	0.01	0.8002	415	0.7955	0.0047
**23**	12	4,4,4	67.49, 109.35, 177.16	0.4	0.05	0.7997	415	0.7976	0.0021
**24**	12	4,4,4	70.64, 114.44, 185.42	0.4	0.1	0.8003	415	0.7991	0.0012
**25**	12	6,4,2	47.16, 104.03, 229.47	0.7	0.01	0.8002	238	0.7915	0.0088
**26**	12	6,4,2	57.67, 127.2, 280.59	0.7	0.05	0.7997	238	0.7938	0.0059
**27**	12	6,4,2	60.36, 133.13, 293.67	0.7	0.1	0.8003	238	0.7962	0.0040
**28**	12	4,4,4	30.38, 74.83, 184.29	0.7	0.01	0.8003	415	0.7891	0.0112
**29**	12	4,4,4	37.15, 91.5, 225.35	0.7	0.05	0.7997	415	0.7996	0.0001
**30**	12	4,4,4	38.88, 95.76, 235.85	0.7	0.1	0.8003	415	0.7901	0.0101
**31**	12	6,4,2	31.45, 95.95, 292.77	1	0.01	0.8002	238	0.7799	0.0203

**32**	12	6,4,2	38.45, 117.33, 357.99	1	0.05	0.7997	238	0.7951	0.0046
**33**	12	6,4,2	40.24, 122.79, 374.68	1	0.1	0.8003	238	0.7915	0.0087
**34**	12	6,4,2	18.96, 82.32, 357.49	1.3	0.01	0.8002	238	0.7694	0.0308
**35**	12	6,4,2	23.18, 100.66, 437.14	1.3	0.05	0.7997	238	0.7868	0.0129
**36**	12	6,4,2	24.26, 105.35, 457.51	1.3	0.1	0.8002	238	0.7915	0.0087
**37**	24	18,6	34.44, 74.68	0.4	0.01	0.8029	84	0.7981	0.0048
**38**	24	18,6	42.57, 92.3	0.4	0.05	0.7994	84	0.795	0.0044
**39**	24	18,6	46.44, 100.69	0.4	0.1	0.8044	84	0.7995	0.0049
**40**	24	12,12	27.07, 61.93	0.4	0.01	0.8028	231	0.8043	-0.0015
**41**	24	12,12	33.46, 76.54	0.4	0.05	0.7995	231	0.794	0.0054
**42**	24	12,12	36.51, 83.49	0.4	0.1	0.8043	231	0.7981	0.0062
**43**	24	18,6	26.89, 97.32	0.7	0.01	0.8028	84	0.7897	0.0131
**44**	24	18,6	33.24, 120.28	0.7	0.05	0.7995	84	0.7888	0.0107
**45**	24	18,6	36.26, 131.21	0.7	0.1	0.8042	84	0.7991	0.0052
**46**	24	12,12	14.01, 74.99	0.7	0.01	0.8028	231	0.7888	0.0140
**47**	24	12,12	17.31, 92.69	0.7	0.05	0.7995	231	0.7907	0.0088
**48**	24	12,12	18.88, 101.12	0.7	0.1	0.8043	231	0.7933	0.0110
**49**	24	18,6	19.35, 119.95	1	0.01	0.8028	84	0.7793	0.0235
**50**	24	18,6	23.91, 148.26	1	0.05	0.7994	84	0.7856	0.0138
**51**	24	18,6	26.09, 161.73	1	0.1	0.8044	84	0.7979	0.0065
**52**	24	18,6	11.8, 142.59	1.3	0.01	0.8011	84	0.7673	0.0338
**53**	24	18,6	14.59, 176.23	1.3	0.05	0.7994	84	0.7796	0.0197
**54**	24	18,6	15.91, 192.26	1.3	0.1	0.8042	84	0.7943	0.0099
**55**	24	12,8,4	30.22, 48.79, 78.76	0.4	0.01	0.8027	4011	0.7987	0.0040
**56**	24	12,8,4	37.35, 60.3, 97.34	0.4	0.05	0.8068	4011	0.8	0.0069
**57**	24	12,8,4	40.75, 65.78, 106.19	0.4	0.1	0.8043	4011	0.8011	0.0033
**58**	24	8,8,8	25.07, 41.09, 67.34	0.4	0.01	0.8028	8623	0.7991	0.0037
**59**	24	8,8,8	30.99, 50.78, 83.23	0.4	0.05	0.7996	8623	0.7931	0.0065
**60**	24	8,8,8	33.8, 55.4, 90.8	0.4	0.1	0.8043	8623	0.7994	0.0049
**61**	24	12,8,4	21.33, 47.84, 107.32	0.7	0.01	0.8029	4011	0.7928	0.0101
**62**	24	12,8,4	26.36, 59.13, 132.64	0.7	0.05	0.7995	4011	0.7898	0.0096
**63**	24	12,8,4	28.76, 64.51, 144.7	0.7	0.1	0.8042	4011	0.7976	0.0066
**64**	24	8,8,8	13.49, 34.05, 85.96	0.7	0.01	0.8028	8623	0.7867	0.0161
**65**	24	8,8,8	16.67, 42.09, 106.24	0.7	0.05	0.7995	8623	0.7867	0.0127
**66**	24	8,8,8	18.19, 45.91, 115.9	0.7	0.1	0.8043	8623	0.7957	0.0086
**67**	24	12,8,4	14.02, 43.86, 137.23	1	0.01	0.8027	4011	0.7833	0.0195

**68**	24	12,8,4	17.33, 54.21, 169.61	1	0.05	0.7995	4011	0.7869	0.0126
**69**	24	12,8,4	18.9, 59.14, 185.02	1	0.1	0.8043	4011	0.7911	0.0132
**70**	24	12,8,4	8.26, 37.22, 167.78	1.3	0.01	0.8027	4011	0.7648	0.0380
**71**	24	12,8,4	10.21, 46.01, 207.36	1.3	0.05	0.7994	4011	0.7815	0.0179
**72**	24	12,8,4	11.14, 50.19, 226.21	1.3	0.1	0.8043	4011	0.795	0.0093
**73**	48	36,12	16.2, 35.4	0.4	0.01	0.8036	455	0.7959	0.0077
**74**	48	36,12	19.29, 42.14	0.4	0.05	0.7995	455	0.7935	0.0060
**75**	48	36,12	21.6, 47.2	0.4	0.1	0.8035	455	0.8017	0.0017
**76**	48	24,24	12.69, 29.31	0.4	0.01	0.8036	1469	0.7971	0.0065
**77**	48	24,24	15.1, 34.9	0.4	0.05	0.7993	1469	0.7891	0.0102
**78**	48	24,24	16.92, 39.08	0.4	0.1	0.8035	1469	0.7957	0.0078
**79**	48	36,12	12.6, 46.19	0.7	0.01	0.8036	455	0.7893	0.0143
**80**	48	36,12	15, 54.99	0.7	0.05	0.7993	455	0.7847	0.0146
**81**	48	36,12	16.8, 61.59	0.7	0.1	0.8035	455	0.7911	0.0123
**82**	48	24,24	6.45, 35.55	0.7	0.01	0.8036	1469	0.7856	0.0180
**83**	48	24,24	7.68, 42.32	0.7	0.05	0.7993	1469	0.7795	0.0168
**84**	48	24,24	8.61, 47.39	0.7	0.1	0.8035	1469	0.7939	0.0096
**85**	48	36,12	9, 56.99	1	0.01	0.8036	455	0.7854	0.0181
**86**	48	36,12	10.72, 67.85	1	0.05	0.7992	455	0.7831	0.0160
**87**	48	36,12	12, 75.99	1	0.1	0.8035	455	0.79	0.0134
**88**	48	36,12	5.4, 67.79	1.3	0.01	0.8036	455	0.76	0.0436
**89**	48	36,12	6.43, 80.7	1.3	0.05	0.7995	455	0.7631	0.0364
**90**	48	36,12	7.2, 90.39	1.3	0.1	0.8035	455	0.7845	0.0190
**91**	48	24,16,8	14.2, 23.03, 37.35	0.4	0.01	0.8037	113949	0.7924	0.0114
**92**	48	24,16,8	16.9, 27.41, 44.47	0.4	0.05	0.7992	113949	0.7847	0.0145
**93**	48	24,16,8	18.93, 30.7, 49.81	0.4	0.1	0.8036	113949	0.7918	0.0118
**94**	48	16,16,16	11.74, 19.35, 31.9	0.4	0.01	0.8036	289981	0.7927	0.0109
**95**	48	16,16,16	13.98, 23.04, 37.98	0.4	0.05	0.7996	289981	0.7924	0.0072
**96**	48	16,16,16	15.66, 25.81, 42.54	0.4	0.1	0.8035	289981	0.7918	0.0117
**97**	48	24,16,8	9.97, 22.55, 51	0.7	0.01	0.8034	113949	0.7802	0.0232
**98**	48	24,16,8	11.87, 26.84, 60.71	0.7	0.05	0.7992	113949	0.7834	0.0158
**99**	48	24,16,8	13.29, 30.06, 67.99	0.7	0.1	0.8035	113949	0.7897	0.0138
**100**	48	16,16,16	6.25, 15.96, 40.79	0.7	0.01	0.8036	289981	0.788	0.0156
**101**	48	16,16,16	7.44, 19, 48.56	0.7	0.05	0.7993	289981	0.783	0.0164
**102**	48	16,16,16	8.33, 21.28, 54.39	0.7	0.1	0.8037	289981	0.7852	0.0185
**103**	48	24,16,8	6.5, 20.61, 65.27	1	0.01	0.8034	113949	0.7817	0.0217

**104**	48	24,16,8	7.74, 24.53, 77.71	1	0.05	0.7994	113949	0.7742	0.0252
**105**	48	24,16,8	8.67, 27.47, 87.03	1	0.1	0.8033	113949	0.7865	0.0167
**106**	48	24,16,8	3.79, 17.39, 79.85	1.3	0.01	0.8037	113949	0.7659	0.0378
**107**	48	24,16,8	4.51, 20.7, 95.06	1.3	0.05	0.7992	113949	0.7654	0.0338
**108**	48	24,16,8	5.05, 23.19, 106.47	1.3	0.1	0.8035	113949	0.7799	0.0236

S.2 Cluster size re-distribution Calculation

Let us define:

(1) The average cluster size from SWSamp package in R is n_e
(2) Small, medium and large cluster sizes are n_s, n_m, n_l.
(3) The number of small, medium and large clusters are: S, M, L
(4) The standard deviation of cluster sizes is: σ

Case 1: Only have small and large clusters

$$CV = \frac{\sigma}{n_e} = \frac{\sqrt{\frac{\sum_S (n_s - n_e)^2 + \sum_L (n_l - n_e)^2}{n_s + n_m + n_l - 1}}}{n_e} \quad (1)$$

$$n_e = (n_s \times S + n_l \times L)/(n_s + n_l) \quad (2)$$

Solve (1) and (2) to get n_s and n_l

Case 2: Have small, medium and large clusters

$$CV = \frac{\sigma}{n_e} = \frac{\sqrt{(\sum_S (n_s - n_e)^2 + \sum_M (n_m - n_e)^2 + \sum_L (n_l - n_e)^2)/(n_s + n_m + n_l - 1)}}{n_e} \quad (3)$$

$$n_e = (n_s \times S + n_m \times M + n_l \times L)/(n_s + n_m + n_l) \quad (4)$$

$$n_m{}^2 = n_s \times n_l \quad (5)$$

Solve (3), (4) and (5) use the nleqslv package in R to get n_s, n_m, n_l

S.3.1 The probability that the attained power falls more than five percent below the nominal (left set of panels) or the expected (right set of panels) power by ICC. The risks decreased as ICC increased in all scenarios, except for the ones with 12 clusters. Panel labels identify the total number of clusters (first line) and the distribution of clusters to the cluster size groups S:L or S:M:L (second line).

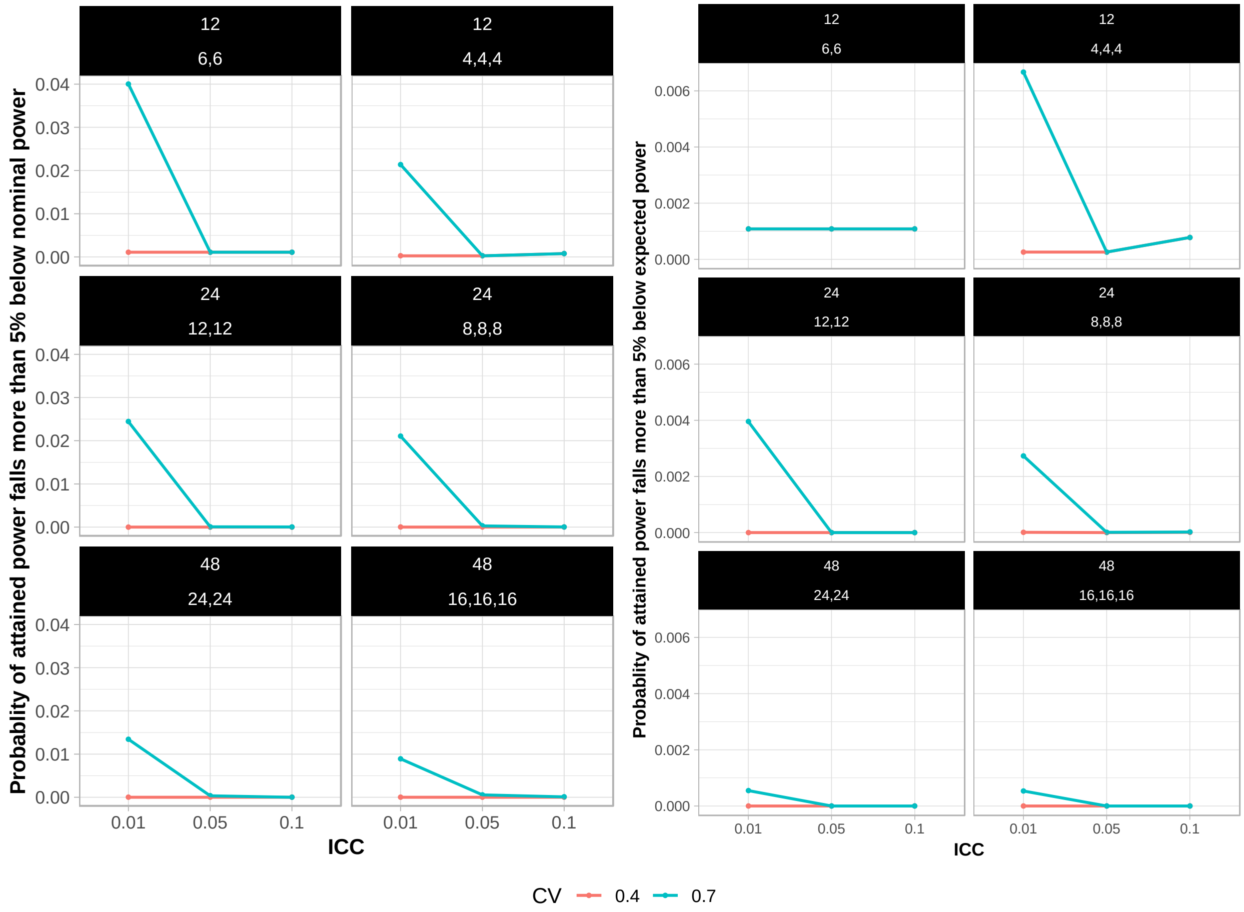

S.3.2 The probability that the attained power falls more than five percent below the nominal (left set of panels) or the expected (right set of panels) power by CV. The risks increased as CV increased in all scenarios except the ones with 12 clusters. The risks were near zero when the CV was smaller than 0.7, except for the scenarios with 12 clusters and two size groups. Panel labels identify the total number of clusters (first line) and the distribution of clusters to the cluster size groups S:L or S:M:L (second line).

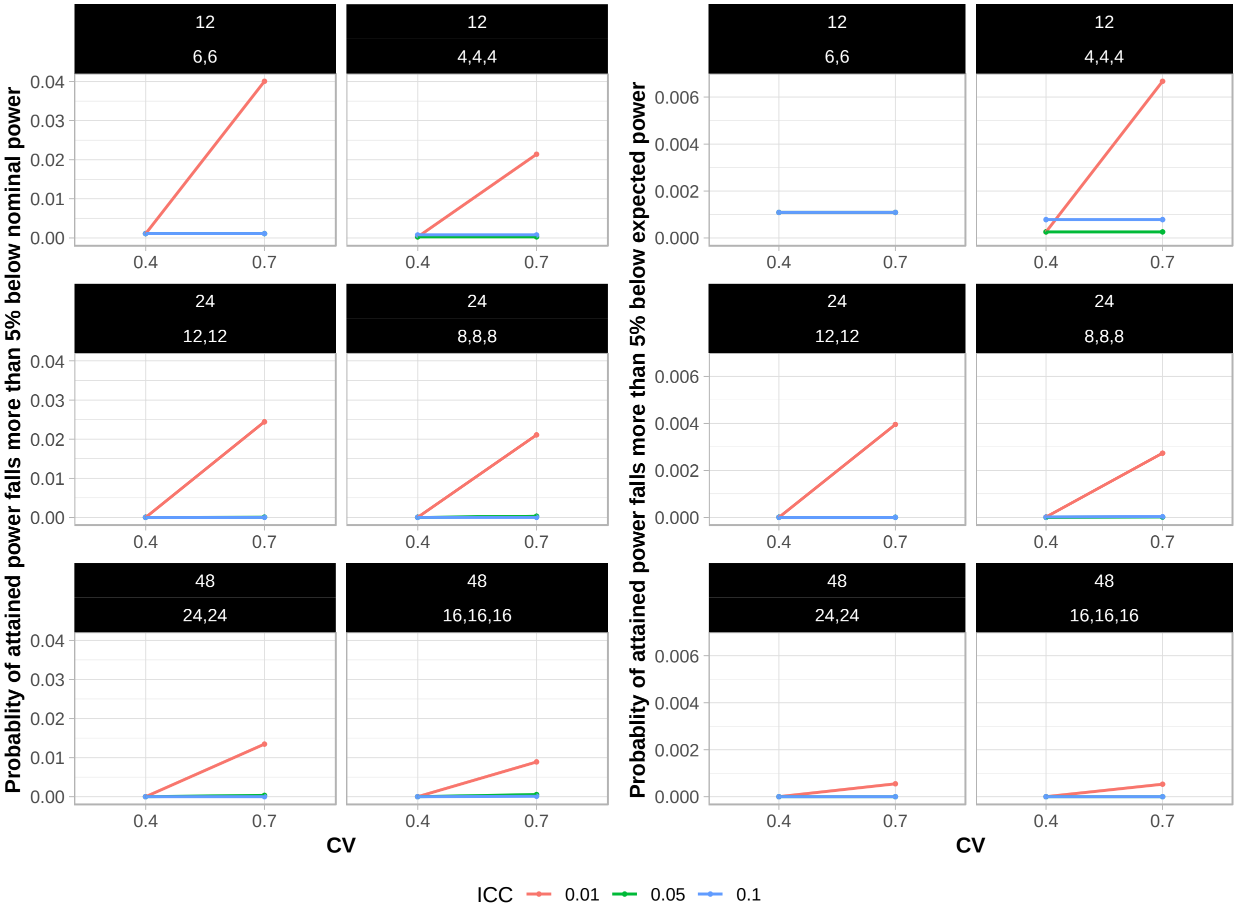

S.4.1 The relationship between treatment-vs-time period correlation and attained power among all 108 scenarios. The attained power decreases considerably as the treatment-vs-time period correlation increases.

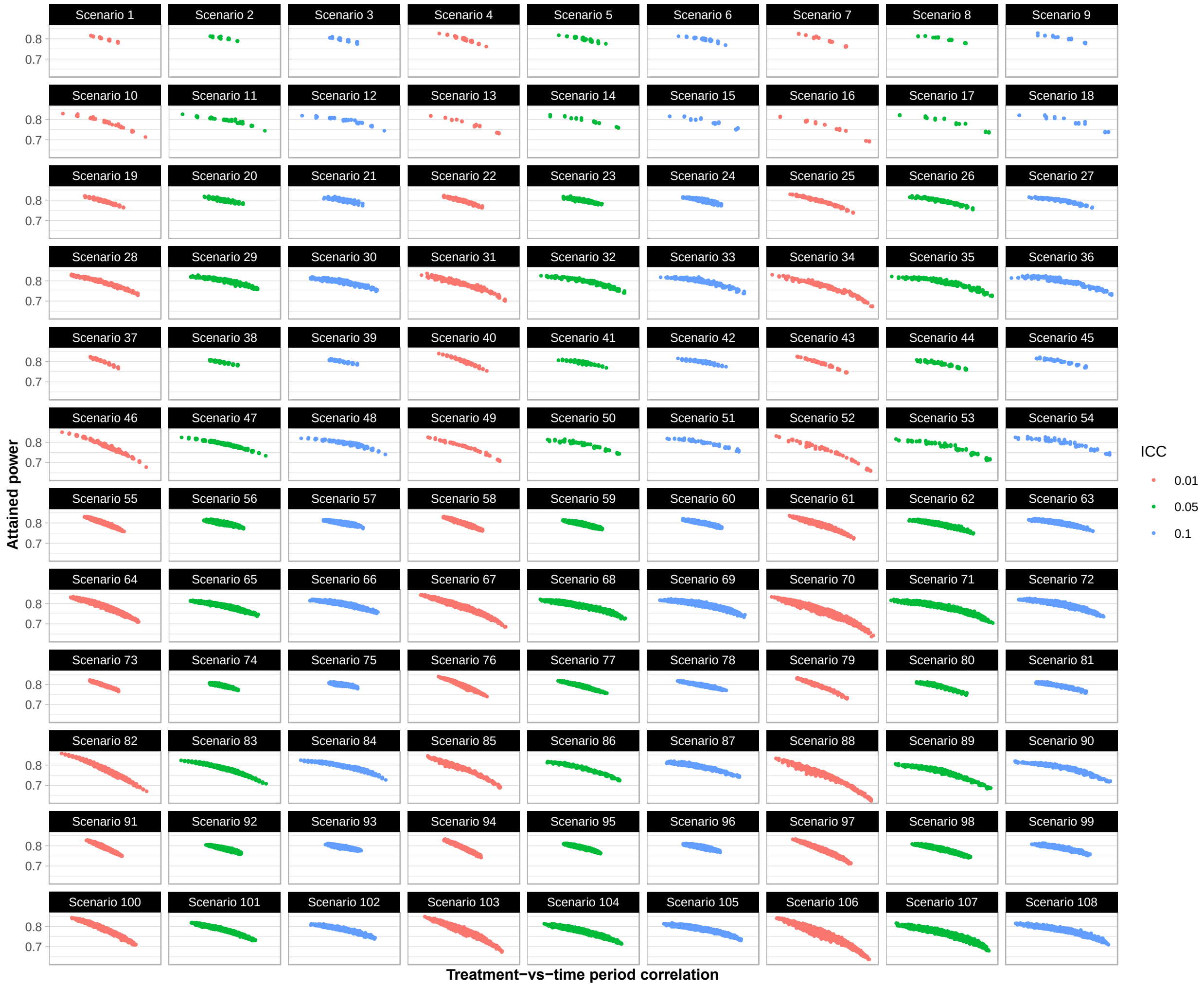

S.4.2 The relationship between absolute treatment group imbalance and attained powers among all scenarios. A triangle pattern was observed in most scenarios. Large treatment group imbalances appeared to be associated with higher power.

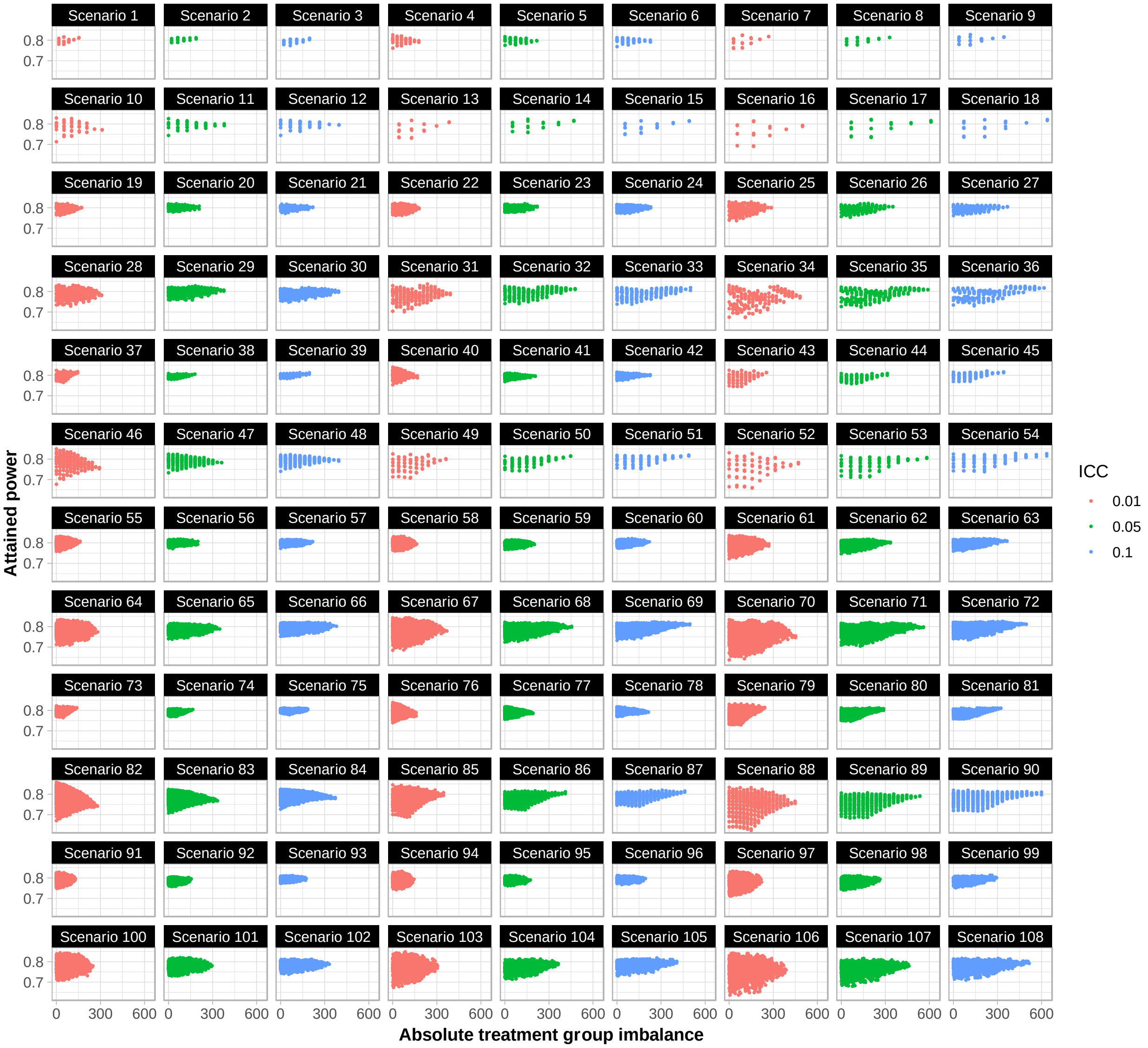

S.5 Distribution of the coefficient for TGI before and after adjusting for TTC. Prior to adjustment, increasing TGI is associated with higher attained power in 90% of the scenarios. After adjustment, the direction of the association between TGI and attained power is inconsistent across the scenarios.

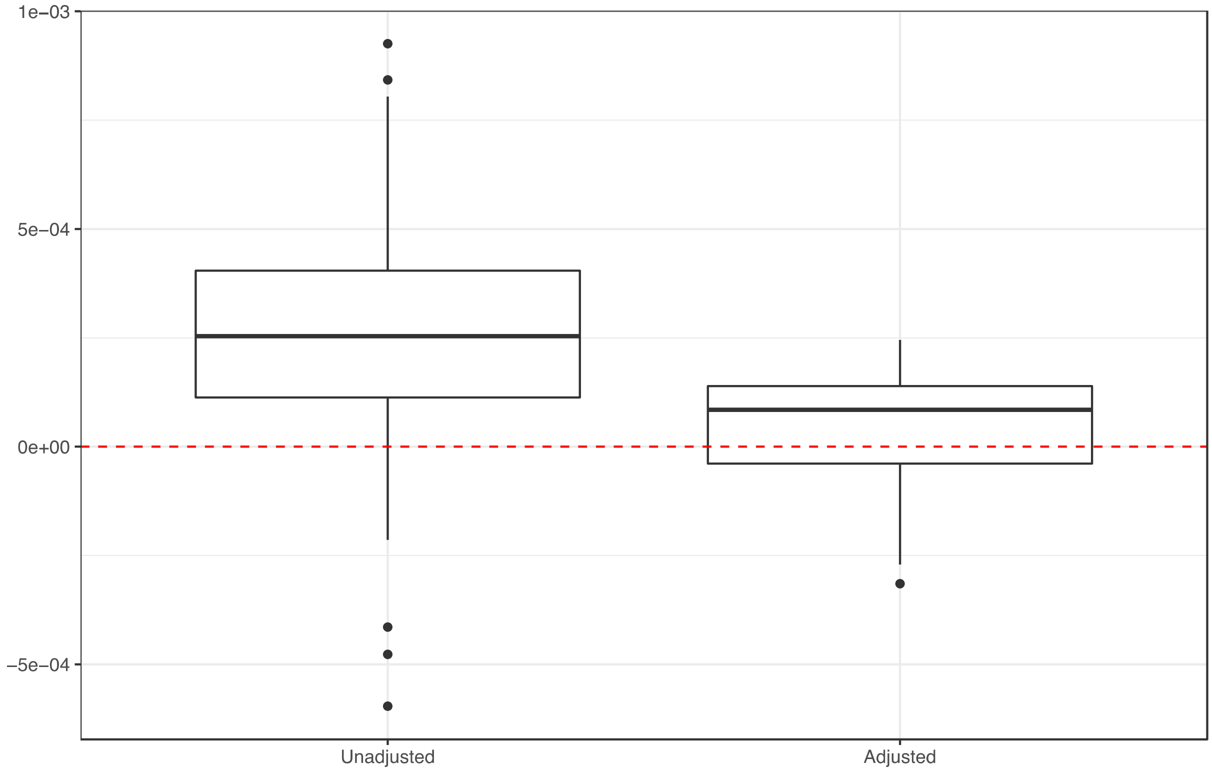

S.6 The RMSPE, maximum absolute prediction error, and average absolute prediction error for all scenarios

Case	Maximum absolute prediction error	RMSPE	Average absolute residual error
**1**	0.004	0.003	0.002
**2**	0.006	0.003	0.002
**3**	0.007	0.005	0.003
**4**	0.007	0.003	0.002
**5**	0.007	0.004	0.003
**6**	0.006	0.003	0.002
**7**	0.006	0.003	0.002
**8**	0.003	0.002	0.001
**9**	0.007	0.004	0.002
**10**	0.008	0.003	0.002
**11**	0.008	0.004	0.002
**12**	0.006	0.003	0.002
**13**	0.007	0.003	0.002
**14**	0.005	0.004	0.003
**15**	0.005	0.005	0.002
**16**	0.006	0.004	0.003
**17**	0.007	0.004	0.003
**18**	0.01	0.004	0.002
**19**	0.007	0.003	0.002
**20**	0.01	0.004	0.003
**21**	0.012	0.004	0.003
**22**	0.009	0.003	0.002
**23**	0.011	0.003	0.002
**24**	0.011	0.004	0.003
**25**	0.009	0.003	0.002
**26**	0.009	0.003	0.002
**27**	0.008	0.003	0.002
**28**	0.011	0.003	0.003

**29**	0.015	0.004	0.003
**30**	0.01	0.004	0.003
**31**	0.017	0.006	0.005
**32**	0.008	0.003	0.003
**33**	0.012	0.004	0.003
**34**	0.015	0.005	0.004
**35**	0.012	0.004	0.003
**36**	0.008	0.003	0.002
**37**	0.005	0.002	0.002
**38**	0.006	0.002	0.002
**39**	0.005	0.003	0.002
**40**	0.008	0.002	0.002
**41**	0.009	0.003	0.002
**42**	0.008	0.003	0.002
**43**	0.006	0.002	0.002
**44**	0.008	0.003	0.002
**45**	0.006	0.002	0.002
**46**	0.009	0.003	0.002
**47**	0.007	0.002	0.002
**48**	0.007	0.003	0.002
**49**	0.008	0.002	0.002
**50**	0.008	0.003	0.002
**51**	0.008	0.003	0.002
**52**	0.01	0.004	0.003
**53**	0.01	0.004	0.003
**54**	0.008	0.004	0.003
**55**	0.009	0.003	0.002
**56**	0.011	0.004	0.003
**57**	0.01	0.003	0.003
**58**	0.01	0.003	0.002

**59**	0.013	0.003	0.002
**60**	0.008	0.003	0.002
**61**	0.011	0.003	0.003
**62**	0.013	0.003	0.002
**63**	0.009	0.002	0.002
**64**	0.013	0.004	0.003
**65**	0.012	0.003	0.002
**66**	0.01	0.003	0.002
**67**	0.013	0.004	0.003
**68**	0.011	0.004	0.003
**69**	0.015	0.004	0.003
**70**	0.021	0.007	0.005
**71**	0.014	0.004	0.003
**72**	0.013	0.003	0.003
**73**	0.006	0.002	0.002
**74**	0.008	0.003	0.002
**75**	0.009	0.003	0.002
**76**	0.011	0.003	0.002
**77**	0.008	0.002	0.002
**78**	0.008	0.002	0.002
**79**	0.009	0.002	0.002
**80**	0.01	0.003	0.002
**81**	0.009	0.003	0.002
**82**	0.009	0.003	0.002
**83**	0.008	0.003	0.002
**84**	0.009	0.003	0.002
**85**	0.011	0.003	0.002
**86**	0.007	0.003	0.002
**87**	0.011	0.004	0.003
**88**	0.019	0.005	0.004

**89**	0.01	0.003	0.003
**90**	0.01	0.003	0.003
**91**	0.009	0.002	0.002
**92**	0.011	0.003	0.002
**93**	0.012	0.003	0.002
**94**	0.01	0.003	0.002
**95**	0.01	0.003	0.002
**96**	0.011	0.003	0.003
**97**	0.011	0.003	0.003
**98**	0.013	0.003	0.003
**99**	0.01	0.003	0.002
**100**	0.014	0.004	0.003
**101**	0.011	0.003	0.002
**102**	0.011	0.003	0.002
**103**	0.016	0.005	0.004
**104**	0.012	0.004	0.003
**105**	0.011	0.004	0.003
**106**	0.019	0.005	0.004
**107**	0.016	0.005	0.004
**108**	0.017	0.004	0.003

S.7 Side-by-side violin plot showing the distribution of difference between the predicted and simulated attained powers for the allocations not used in model fitting for scenario #64 and #103

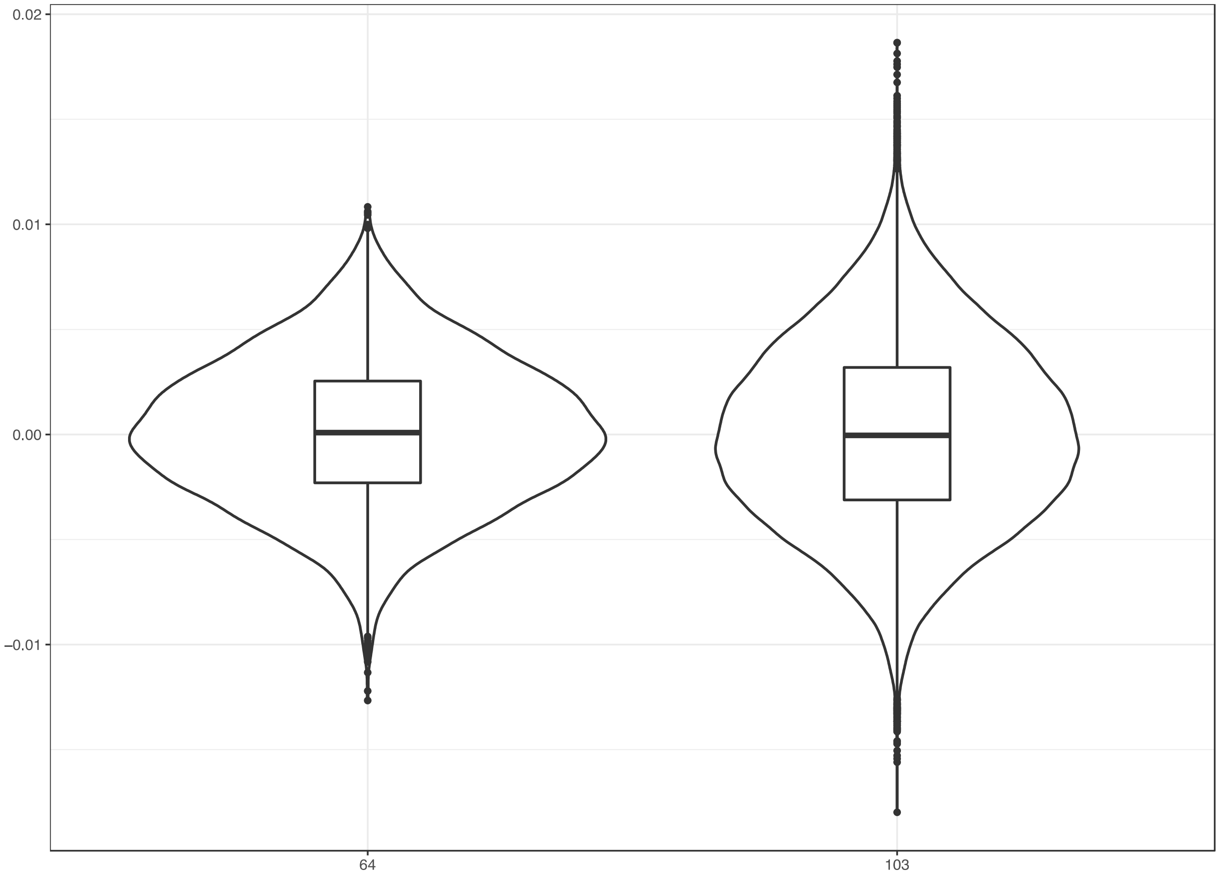

Supplement: Supplementary file 1 — Additional file 1: S.1 List of all evaluated scenarios. S.2 Cluster size re-distribution Calculation. S.3.1 Risk of obtaining low vs ICC for scenarios with equal distribution of clusters. S.3.2 Risk of obtaining low vs CV for scenarios with equal distribution of clusters. S.4.1 The relationship between TTC and attained power for all scenarios. S.4.2 The relationship between TGI and attained power for all scenarios. S.5 Distribution of the coefficient for TGI before and after adjusting for TTC. S.6 The RMSPE, maximum absolute prediction error, and average absolute prediction error for all scenarios. S.7 Side-by-side violin plot showing the distribution of difference between the predicted and simulated attained powers for the allocations not used in model fitting for scenario #64 and #103. [file 12874_2020_1036_MOESM1_ESM.pdf]
